# Case Report: Contrast adenosine stress echocardiography revealing microvascular dysfunction in cardiac AL amyloidosis

**DOI:** 10.3389/fcvm.2026.1710211

**Published:** 2026-02-05

**Authors:** Yuqian Dai, Yan Deng, Ni Lin, Feifei Che, Chunmei Li, Lixue Yin

**Affiliations:** 1 Department of Anatomical Sciences, School of Medicine, St. George’s University, St. George's, Grenada; 2 Department of Cardiovascular Ultrasound & Noninvasive Cardiology, Sichuan Provincial People’s, Hospital, University of Electronic Science and Technology of China, Chengdu, China; 3 Ultrasound Medicine and Computational Cardiology Key Laboratory of Sichuan Province, Sichuan Provincial People’s Hospital, University of Electronic Science and Technology of China, Chengdu, China; 4 Department of Hematology, Sichuan Provincial People’s Hospital, University of Electronic Science and Technology of China, Chengdu, China

**Keywords:** cardiac amyloidosis, case report, contrast stress echocardiography, infiltrative cardiomyopathy, multiple myeloma

## Abstract

Cardiac amyloidosis (CA) is a rare but increasingly recognized cause of restrictive cardiomyopathy. Noninvasive assessment of coronary microvascular dysfunction in CA can facilitate earlier diagnosis and improve disease monitoring. We present a case of light-chain (AL) cardiac amyloidosis manifesting as exertional angina with non-obstructive epicardial coronary arteries, in which contrast adenosine stress echocardiography combined with Doppler-derived coronary flow velocity reserve (CFVR) measurement revealed impaired myocardial perfusion and reduced CFVR. The findings were consistent with cardiac magnetic resonance (CMR) and laboratory results, confirming the diagnosis of CA. This case demonstrates the clinical application of contrast adenosine stress echocardiography as a practical adjunct to advanced imaging modalities for evaluating microvascular dysfunction in CA.

## Introduction

Cardiac amyloidosis (CA) is an underdiagnosed cause of restrictive cardiomyopathy, resulting from extracellular deposition of misfolded amyloid fibrils. The most common subtypes are immunoglobulin light-chain (AL) amyloidosis and transthyretin (ATTR) amyloidosis (1). AL-CA often progresses rapidly, with a median survival of less than one year once heart failure develops.

While typical presentations include heart failure symptoms, arrhythmias, and restrictive filling on echocardiography, ischemic-type chest pain with normal or minimally diseased epicardial coronary arteries is a less common but recognized feature. This may reflect amyloid infiltration of small intramyocardial vessels, causing microvascular dysfunction and myocardial ischemia. Previous echocardiographic studies, such as that by Clemmensen et al. (2), have investigated Coronary Flow Velocity Reserve (CFVR) in cardiac amyloidosis using exercise-based protocols. Building on this foundation, our case highlights the clinical utility of contrast adenosine stress echocardiography with CFVR measurement as a practical, physiologic approach for microvascular evaluation.

We report a case of AL-CA presenting with exertional angina and non-obstructive epicardial coronaries, in which contrast adenosine stress echocardiography demonstrated decreased myocardial perfusion and impaired coronary flow reserve, contributing to early diagnosis and treatment.

## Case presentation

A 59-year-old man was admitted with recurrent exertional chest pain. One year prior, he had developed intermittent chest discomfort relieved by rest. In May 2022, he visited a regional hospital and his coronary angiography revealed approximately 20% stenosis of the right coronary artery, with no significant abnormalities in the left main, left anterior descending (LAD), or left circumflex arteries.

Six months later, chest computed tomography for recurrent pain showed a moderate right pleural effusion. Subsequent examinations led to a diagnosis of pulmonary tuberculosis and treated with rifampicin, ethambutol, and isoniazid. Despite therapy, angina worsened in both intensity and duration, and bilateral lower limb edema developed one week prior to admission in March 2024.

Past medical history was significant for hypertension for over 10 years, previously well-controlled with triple therapy (Sacubitril Valsartan Sodium 100 mg qd, Metoprolol 23.75 mg qd, Amlodipine 2.5 mg qd) until 2023, when his blood pressure became abnormally low. Sacubitril/valsartan was prescribed according to current Chinese guidelines, which recognize its efficacy in the treatment of primary hypertension. After withdrawal of antihypertensive medicine, he maintained normal blood pressure. Family history was unremarkable.

Physical examination was notable for mild bilateral lower limb edema. Vital signs were stable: blood pressure 101/55–128/83 mmHg, heart rate 55–78 bpm. Heart sounds were normal without murmurs, rubs, or gallops. There was no jugular venous distension. The remainder of the examination was unremarkable.

Electrocardiography (ECG) showed regular sinus rhythm at 67 bpm, poor R-wave progression in V1–V3, ST-segment depression in V4–V6, and normal QRS voltages.

Laboratory tests revealed an elevated B-type natriuretic peptide (BNP) level of 3,157 pg/mL (normal value <100 pg/mL) and a high-sensitivity cardiac troponin T(hs-cTnT) level of 135 ng/L (normal value <16.8 ng/L). Complete blood cell counts, blood electrolyte, kidney and liver function tests were normal.

**Figure 1:**
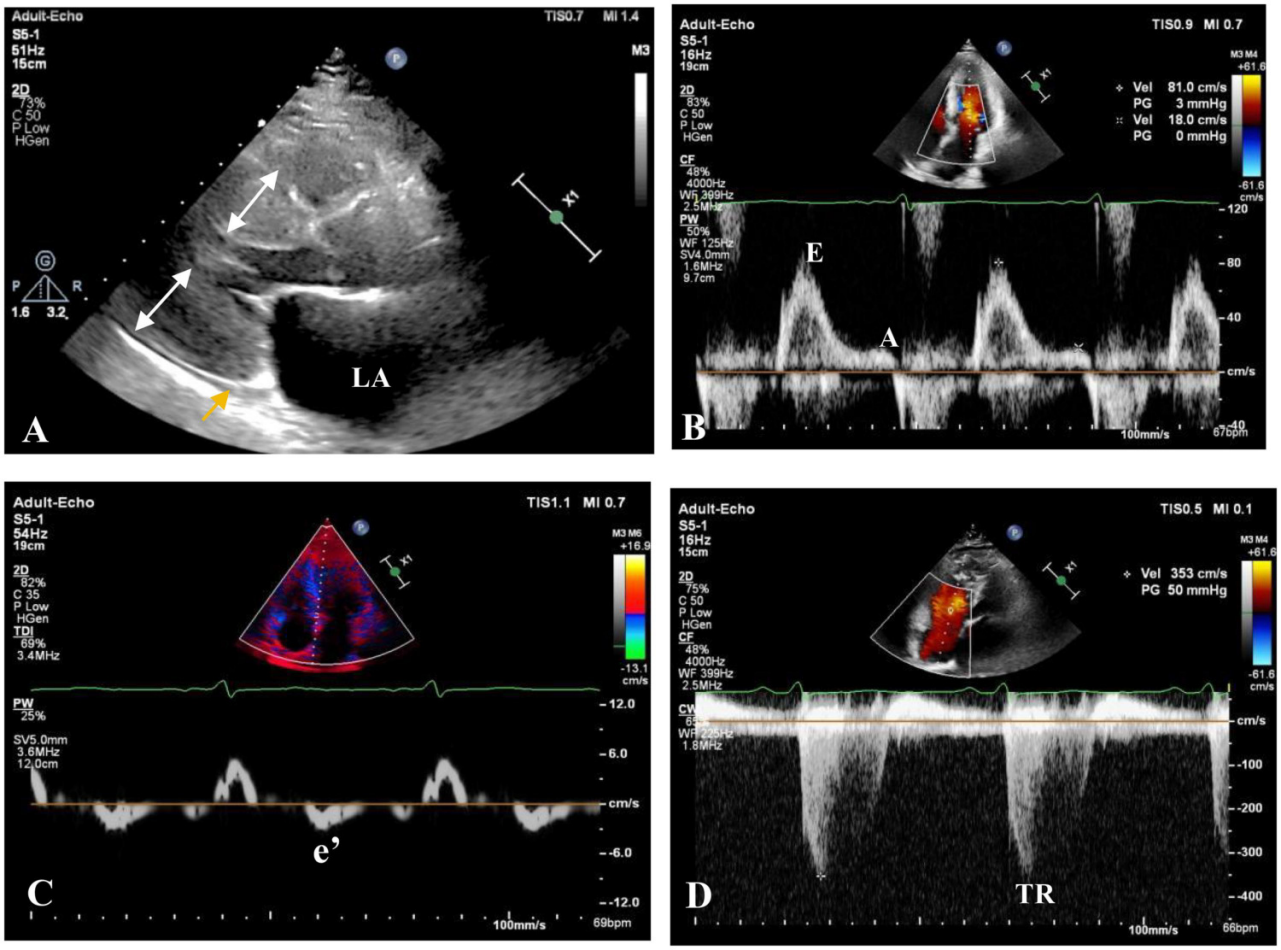
Transthoracic echocardiogram showing typical echocardiographic features of cardiac amyloidosis: **(A)** parasternal long-axis view showing severe concentric left ventricular hypertrophy (white arrow), sparkling granular appearance of the myocardium, minimal pericardial effusion (yellow arrow), and left atrial(LA) enlargement. **(B)** Transmitral valve inflow diastolic peak early (E 0.82 m/s)/later (A 0.18 m/s) velocity obviously increased. **(C)** Tissue Doppler imaging showing markedly reduced septal early diastolic velocity of the mitral valve annular (e′ 3 cm/s). **(D)** Elevated tricuspid regurgitation velocity (TR 3.53 m/s) consistent with pulmonary hypertension.

Transthoracic echocardiography (Figure 1) demonstrated severe concentric left ventricular (LV) hypertrophy (wall thickness 16–19 mm) with increased echogenicity, a normal LV cavity size (end-diastolic diameter 44 mm), and preserved systolic function (ejection fraction 70%, Simpson's method). Diastolic function was severely impaired (grade 3) with a restrictive filling pattern (E/A ratio 4.56, septal e' 3 cm/s, E/e' ratio 27). The right ventricle was normal in size and wall thickness but showed mild systolic dysfunction (TDI s' 8 cm/s, TAPSE 13 mm). There was biatrial enlargement (left atrium 53 × 65 × 58 mm, right atrium 55 × 51 mm) with minimal phasic change, mild mitral regurgitation, mild-moderate tricuspid regurgitation, and associated pulmonary hypertension (estimated PASP 60 mmHg). The inferior vena cava was normal in diameter (17 mm) with reduced inspiratory collapse (estimated RAP 8 mmHg), and a minimal pericardial effusion was present.

To evaluate for microvascular dysfunction as a cause of his chest pain, we performed myocardial contrast echocardiography (MCE) with adenosine stress echocardiography (SE) and CFVR assessment. The blood flow spectrum of the LAD at the mid part was obtained in a modified apical view using pulsed-wave Doppler (PW) under the guidance of color Doppler flow mapping. MCE was performed in apical four-chamber, two-chamber, and three-chamber views during a continuous infusion of ultrasound contrast agent SonoVue (Bracco Suisse SA, Switzerland). Real-time cine images were acquired for up to 15 cardiac cycles after a high-mechanical index (MI) “flash” to assess replenishment. After baseline imaging and coronary flow velocity recording, intravenous adenosine (140 μg/kg/min) was infused for 6 min. Hyperemic flow and MCE images were acquired during the third minute of infusion. CFVR was calculated as the ratio of hyperemic to baseline average peak and mean diastolic flow velocities over 3 cycles; a value <2 was considered abnormal. Brief high-MI impulses were administered to clear myocardial contrast, and then myocardial perfusion was analyzed visually, with normal replenishment defined as within 5 s at rest and 2 s during adenosine-induced hyperemia.

**Figure 2:**
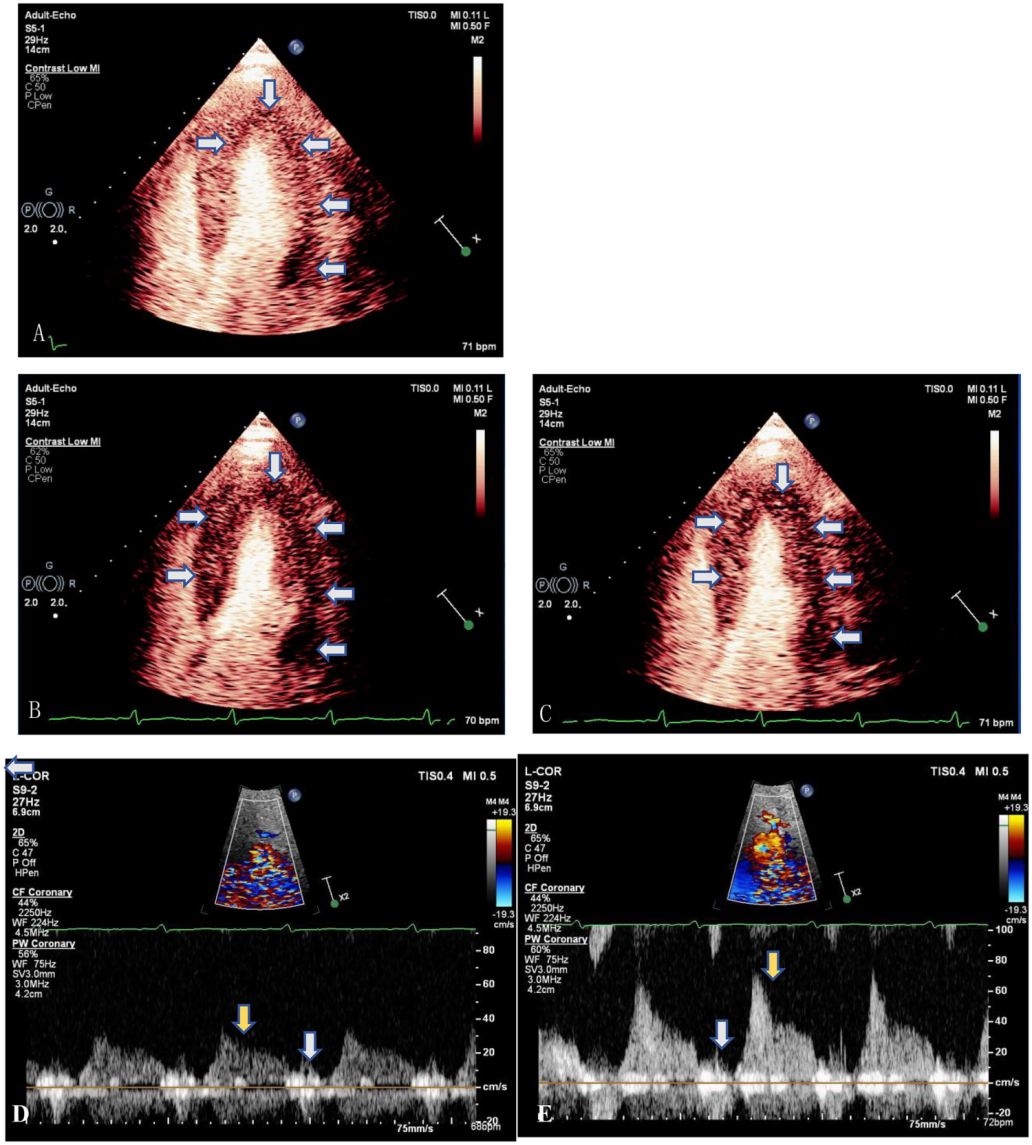
Myocardial contrast echocardiography and coronary blood flow velocity assessment before and after adenosine infusion, consistent with impaired microvascular function and decreased blood flow reserve. **(A–C)** End-systolic perfusion by myocardial contrast echocardiography in apical four-chamber view. **(A)** Mild perfusion defect of LV part segments (arrows) before high-MI flash impulse. **(B)** Significant reduction in perfusion in the sixth sec post flash (arrows) before adenosine infusion. **(C)** Three minutes after adenosine infusion showing significant reduction in perfusion in the third sec post flash (arrows). **(D,E)** Coronary blood flow velocity spectrum of the mid left anterior descending artery (LAD). **(C)** Before adenosine infusion. **(D)** At the third minute after adenosine infusion. Reduced blood flow is seen with minimal systolic blood flow (white arrow), and increased diastolic blood flow resistance (yellow arrow).

**Figure 3:**
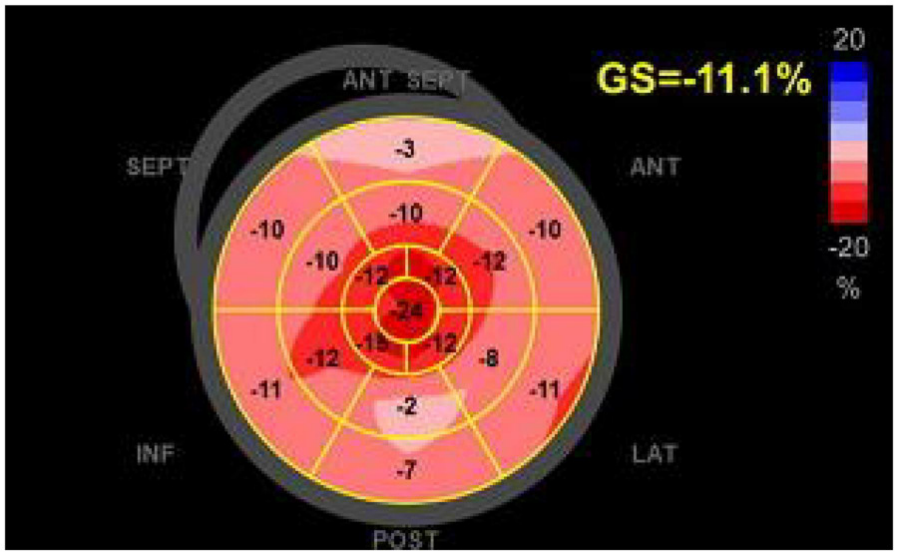
Speckle-tracking strain imaging of left ventricle revealing reduced global longitudinal strain, with relative apical sparing, marked impairment at the base-midventricle (“cherry on the top” pattern), a characteristic feature of cardiac amyloidosis.

The results demonstrated markedly abnormal myocardial perfusion and a significantly reduced CFVR in the mid-LAD, consistent with microvascular dysfunction (Figure 2; Supplementary Video S1 and S2). MCE revealed a mild perfusion defect in most LV segments before the high-MI flash (Figure 2A). Following the flash, replenishment was delayed, showing patchy or absent contrast uptake after 6 s at rest (Figure 2B) and 3 s during adenosine stress (Figure 2C). Coronary flow velocities at rest were: peak diastolic velocity (Rest-Vmax) 0.32 m/s, mean diastolic velocity (Rest-Vmean) 0.20 m/s (Figure 2D). During hyperemia, velocities increased to: peak diastolic velocity (Peak-Vmax) 0.76 m/s, mean diastolic velocity (Peak-Vmean) 0.36 m/s (Figure 2E), yielding a CFVR-Vmax of 2.4 and a CFVR-Vmean of 1.8. The spectral pattern was also abnormal, showing sparse systolic flow, a blunted diastolic upslope, and a high-resistance pattern. The patient tolerated the procedure well without adverse symptoms.

The severity of the LV hypertrophy was disproportionate to the patient's hypertension history, and the discrepancy with normal ECG voltages raised strong suspicion for cardiac amyloidosis. We therefore performed speckle-tracking echocardiography (STE), which revealed a pattern characteristic of CA: severely impaired basal and mid-ventricular segments with relative apical sparing, producing a “cherry-on-the-top” sign on the longitudinal strain bullseye map (Figure 3). It is worth noting that while these echocardiographic findings strongly suggested CA, global longitudinal strain (GLS) analysis was performed after the stress echocardiogram. This sequential approach was taken because the initial clinical focus was on evaluating his angina for microvascular ischemia, and strain analysis is not yet a fully integrated routine in all echocardiographic systems under current Chinese guidelines.

Further workup with cardiac magnetic resonance (CMR) confirmed significant left ventricular hypertrophy with preserved systolic function (ejection fraction 58%), biatrial enlargement, and late gadolinium enhancement involving multiple left ventricular segments, suggestive of an infiltrative cardiomyopathy. Representative CMR images are not included, as they demonstrated nonspecific late gadolinium enhancement without perfusion data, and this report focuses on the echocardiographic techniques. A skeletal MRI survey showed no lytic lesions. Serum immunofixation electrophoresis showed a markedly elevated kappa free light chain concentration (185 mg/L) with a normal lambda level, resulting in abnormal kappa to lambda ratio of 1.9 (reference value 0.26–1.65) and free light chain kappa/lambda ratio of 11.08 (reference 0.31–1.56). Urine electrophoresis identified Bence Jones protein type kappa.

**Figure 4:**
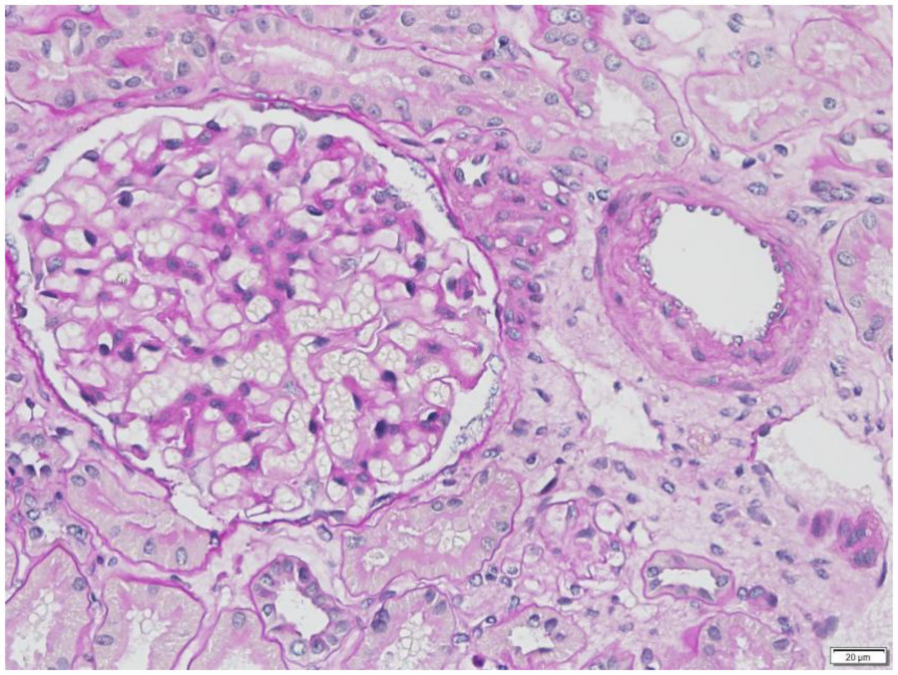
Renal biopsy (PAS stain, ×400) showing amorphous PAS-positive material within glomerular mesangium and small vessel walls, consistent with AL-type amyloid deposition.

A bone marrow biopsy revealed monoclonal kappa-restricted plasma cells (1.95%). A renal biopsy confirmed AL-type amyloid deposition in small arteries, consistent with early amyloid nephropathy (Figure 4). The final diagnosis was systemic AL amyloidosis with cardiac, renal, and bone marrow involvement, Mayo 2004 stage III, 2012 stage III, ECOG score 1.

The patient was initially treated with bortezomib, cyclophosphamide, and dexamethasone for his AL-CA due to financial constraints, alongside guideline-directed diuretic therapy for heart failure. After three cycles, his condition worsened with persistent angina, dyspnea, edema, and rising biomarkers (BNP 14,423 pg/mL, hs-cTnT 234 ng/L). ECG showed V1-4 Q waves, ST-T changes. The bone marrow monoclonal plasma cell ratio increased to 10%. Given this progression, his regimen was changed to daratumumab, bortezomib, and dexamethasone. On subsequent follow-up, his exertional angina and fatigue resolved, and his lower limb edema disappeared. The bone marrow showed no abnormal monoclonal plasma cells, and his serum free light chain kappa/lambda ratio normalized to 2.12. His biomarkers improved (BNP 2,189 pg/mL, hs-cTnT 126 ng/L). Follow-up echocardiography showed no significant changes in cardiac structure and function. The patient achieved hematologic remission with stable organ involvement. Table 1 provides an overview of the patient's admission timeline and the diagnostic workup leading to the diagnosis of cardiac amyloidosis.

**Table 1:** Summary.

Time	Event
Day 0	Patient admitted with history of progressive dyspnea, fatigue, and edema. Transthoracic echocardiography (TTE) performed, suggesting restrictive cardiomyopathy.
Day 1	Myocardial contrast adenosine stress echocardiography demonstrated reduced myocardial perfusion. A decreased coronary flow velocity reserve (CFVR) of the left anterior descending artery (LAD) at 1.8 indicated impaired microvascular function. Speckle-tracking strain imaging of the left ventricle revealed reduced global longitudinal strain with a characteristic apical sparing pattern, raising suspicion for cardiac amyloidosis.
Subsequent days	Additional multimodality evaluation (cardiac MRI and other tests) supported diagnosis of suspected cardiac amyloidosis, and renal biopsy confirmed AL amyloidosis.
Follow-up	Patient managed with hematology/oncology and cardiology teams; ongoing therapy for multiple myeloma and evaluation for amyloidosis-directed therapy.

## Discussion

AL amyloidosis is the second most common form of CA after ATTR, resulting from monoclonal immunoglobulin light chains produced by a clonal plasma cell dyscrasia. The extracellular deposition of amyloid fibrils causes both architectural disruption and proteotoxic injury from soluble precursors, leading to rapidly progressive organ dysfunction. While the heart and kidneys are major targets, any organ can be involved except the brain.

Clinical presentations of AL-CA most often include heart failure with preserved ejection fraction, exertional dyspnea, fatigue, arrhythmias, conduction disturbances, and occasionally ischemic-type chest pain despite unobstructed coronary arteries. Cardiac involvement is present in up to 71% of AL cases at diagnosis and is the major determinant of prognosis (3–5).

Biopsy and autopsy studies (6–8) have demonstrated that amyloid deposition is most pronounced within the myocardium and small intramyocardial vessels, with epicardial coronary arteries typically spared. Amyloidosis causes microvascular dysfunction and ischemia through three overlapping mechanisms: deposition within the walls of small intramyocardial vessels causing intimal thickening and capillary disruption, interstitial deposition causing perivascular compression, and autonomic and/or endothelial dysfunction. High-grade myocardial infiltration (>50% of the myocardium) is frequent in AL amyloidosis, while vascular involvement of small intramural arteries and arterioles is reported in up to 88%–90% of cases, significantly higher than in ATTR amyloidosis.

For patients presenting with chest pain and angina, accurate evidence of myocardial ischemia is crucial for guiding therapy and improving prognosis. Beyond obstructive coronary artery disease, coronary microvascular dysfunction (CMD) caused by various conditions is increasingly recognized. The coronary microvasculature, comprised of pre-arterioles and arterioles <500 μm in diameter, regulates up to 90% of total myocardial blood flow. Although direct imaging of these vessels remains challenging, several modalities, including echocardiography, CMR, single-photon emission computed tomography (SPECT), and positron emission tomography (PET), are commonly used to evaluate microvascular function and play a vital role in diagnosis, treatment, and prognosis.

CMR is highly valuable for detecting CA (9, 10). Multiparametric CMR allows for the assessment of myocardial perfusion and quantification of myocardial blood flow (MBF). A study by Chacko et al. (11) using adenosine perfusion CMR in CA patients demonstrated severe reductions in both rest and stress MBF, as well as in myocardial perfusion reserve (MPR), confirming the presence of myocardial ischemia. This ischemia arises not only from systolic and diastolic dysfunction but also from amyloid infiltration causing epicardial arterial involvement and capillary disruption and rarefaction.

SPECT and PET are other established techniques for perfusion imaging. Dorbala et al. (12) prospectively assessed microvascular dysfunction using N-13 ammonia PET/CT in 21 patients with CA. They found that compared to a control group with hypertensive left ventricular hypertrophy, the CA patients had a significantly higher prevalence of ischemic defects (57%) and transient ischemic LV dilatation (76%). Furthermore, the CA group exhibited significantly lower stress MBF and myocardial flow reserve, alongside significantly higher microvascular resistance.

Contrast echocardiography is approved not only for improving the accuracy of wall motion assessment but also for the simultaneous evaluation of myocardial perfusion during vasodilator stress, primarily used for assessing myocardial ischemia and viability in coronary artery disease. Real-time MCE examines myocardial blood flow and volume; brief high-MI impulses clear myocardial contrast, after which replenishment is analyzed visually or quantitatively.

Large multicenter trials (13) have shown that vasodilator stress MCE has equivalent specificity and superior sensitivity to SPECT for detecting coronary artery disease, using angiography as a reference standard. This may be because MCE has better spatial resolution and assesses both capillary blood volume and velocity—the latter being a more sensitive marker of abnormal perfusion. Compared to SPECT or CMR, contrast adenosine stress echocardiography with Doppler-derived CFVR simultaneously provides information on wall motion, myocardial perfusion, and perfusion reserve in a single exam. Its advantages of rapid performance, wide availability, cost-effectiveness, lack of radiation, and bedside feasibility make it an important tool for evaluating coronary microvascular diseases (14).

In this patient, contrast adenosine stress echocardiography demonstrated markedly delayed myocardial perfusion and a reduced CFVR of 1.8 in the LAD, confirming impaired microvascular function despite non-obstructive epicardial coronaries. Adenosine stress with contrast enhancement allowed direct visualization of perfusion abnormalities, while CFVR provided a quantitative, objective measure of microvascular dysfunction. These findings were concordant with the apical-sparing pattern on GLS and the diffuse late gadolinium enhancement on CMR, together supporting the diagnosis of an infiltrative cardiomyopathy. This multiparametric echocardiographic approach provided direct physiologic evidence of microvascular dysfunction, explaining the patient's ischemic symptoms in the absence of epicardial disease.

Beyond amyloidosis, CFVR and MCE are extensively applied in other conditions associated with CMD, such as hypertrophic cardiomyopathy, heart failure with preserved ejection fraction (HFpEF), diabetic cardiomyopathy, and microvascular angina. However, amyloidosis is distinct due to its infiltrative pathophysiology: extracellular fibril deposition and perivascular compression often leads to diffuse and potentially irreversible microvascular impairment. Combining contrast perfusion imaging with Doppler-derived CFVR in amyloidosis therefore offers complementary insights distinct from the functional microvascular changes seen in other diseases.

A study by Clemmensen et al. (2) evaluated CFVR during semisupine exercise in 27 CA patients and found significantly lower CFVR and GLS compared to healthy controls, with both parameters associated with exercise capacity. Vasodilator stress perfusion imaging may offer advantages over inotropic stress for detecting CMD, including better image quality due to lower heart rates and less translational motion. Reports using combined contrast adenosine stress echocardiography and Doppler-derived CFVR to interrogate microvascular circulation in CA are rare. In addition to reduced CFVR, we also observed abnormal spectral patterns—sparse systolic flow, a blunted diastolic upslope, and a high-resistance pattern—making this case notable for demonstrating comprehensive diagnostic value. Furthermore, whether echocardiographic measures of microvascular function can track treatment response or predict recovery remains largely unexplored and warrants further investigation.

Early and accurate diagnosis of CA is crucial, as it directly impacts therapeutic choices and overall prognosis (15, 16). The early identification of a monoclonal protein in this case prompted targeted biopsies, confirming AL amyloidosis with cardiac and renal involvement. Treatment strategies are guided by cardiac and renal biomarkers. The combination of daratumumab, cyclophosphamide, bortezomib, and dexamethasone is now a standard of care. This patient attained a satisfactory response after switching to daratumumab, bortezomib, and dexamethasone. The outcome reflects three key therapeutic principles (16): (1) rapid, sustained reduction of monoclonal protein production; (2) tailoring regimens to disease extent and organ involvement; and (3) providing organ-directed supportive care to minimize complications and improve quality of life.

While echocardiography combined with CMR is used to diagnose and prognosticate CA, echocardiography remains the cornerstone for initial diagnosis and is often the first investigation to raise suspicion. Several typical morphologic and functional “red flags” should raise suspicion for CA, including: increased LV and RV wall thickness, low ECG voltages relative to wall thickness, restrictive diastolic dysfunction, reduced GLS with apical sparing, and pericardial effusion (17).

This case exhibited most of these echocardiographic features. It highlights the importance of considering CA in patients with disproportionate ECG voltages relative to LV wall thickness, STE showing apical sparing, unexplained angina with non-obstructive coronaries, and perfusion studies suggesting CMD. For clinicians, the key lesson is that systematic investigation in patients with these “red flags” is crucial for the early initiation of specific chemotherapy.

Beyond this individual case, maintaining AL amyloidosis in the differential diagnosis for patients with nephrotic-range proteinuria, unexplained restrictive or non-ischemic cardiomyopathy, hepatomegaly, peripheral neuropathy, or atypical multiple myeloma may expedite diagnosis. Despite advances in imaging and therapy, further studies are needed to validate echocardiography as a tool not only for detecting microvascular dysfunction but also for monitoring treatment response and recovery. This case demonstrates the clinical application of contrast-enhanced stress echocardiography with Doppler-derived CFVR to assess and quantify coronary microvascular dysfunction in cardiac amyloidosis, providing complementary diagnostic insight alongside advanced imaging modalities.

## Limitations

Certain limitations in this case should be acknowledged. First, the gold standard for CA diagnosis is endomyocardial biopsy. While the typical imaging findings and renal biopsy strongly support AL-CA, the patient did not undergo endomyocardial biopsy to directly confirm myocardial and microvascular amyloid deposition. Second, neither the patient nor the responsible physician opted for follow-up contrast adenosine stress echocardiography to assess changes in microvascular function after treatment. Third, myocardial perfusion was analyzed visually rather than with quantitative MCE assessments of blood flow and reserve. Finally, the initiation of daratumumab was delayed due to cost, which may have influenced the patient's early treatment response and clinical course.

## Conclusion

This case illustrates that contrast adenosine stress echocardiography can reveal microvascular dysfunction in cardiac amyloidosis, even when epicardial coronaries are unobstructed. Integrating echocardiographic perfusion and CFVR assessment into the diagnostic pathway may facilitate earlier recognition, guide timely hematologic evaluation, and holds promise for monitoring treatment response in the future.

## Data Availability

The original contributions presented in the study are included in the article/Supplementary Material, further inquiries can be directed to the corresponding author.

## Supplementary material

Supplementary Video S1 Myocardial contrast echocardiography before adenosine infusion, four-chamber apical view, demonstrating a markedly delayed contrast appearance with reduced contrast intensity, suggesting decreased myocardial perfusion velocity and blood volume.

Supplementary Video S2 Myocardial contrast echocardiography three minutes after adenosine infusion, four-chamber apical view, delayed and sparse myocardial perfusion is evident, consistent with microvascular dysfunction and reduced coronary flow reserve.
