# Correction: Case Report: Contrast adenosine stress echocardiography revealing microvascular dysfunction in cardiac AL amyloidosis

**DOI:** 10.3389/fcvm.2026.1805836

**Published:** 2026-03-06

**Authors:** Yuqian Dai, Yan Deng, Ni Lin, Feifei Che, Chunmei Li, Lixue Yin

**Affiliations:** 1 Department of Anatomical Sciences, School of Medicine, St. George’s University, St. George’s, Grenada; 2 Department of Cardiovascular Ultrasound & Noninvasive Cardiology, Sichuan Provincial People’s, Hospital, University of Electronic Science and Technology of China, Chengdu, China; 3 Ultrasound Medicine and Computational Cardiology Key Laboratory of Sichuan Province, Sichuan Provincial People’s Hospital, University of Electronic Science and Technology of China, Chengdu, China; 4 Department of Hematology, Sichuan Provincial People’s Hospital, University of Electronic Science and Technology of China, Chengdu, China

**Keywords:** cardiac amyloidosis, case report, contrast stress echocardiography, infiltrative cardiomyopathy, multiple myeloma

Figure/table caption

There was a mistake in the caption of figure 2 as published. The last two panels in the caption were incorrectly referred to as (C) and (D). The corrected caption of figure 2 appears below.

“Myocardial contrast echocardiography and coronary blood flow velocity assessment before and after adenosine infusion, consistent with impaired microvascular function and decreased blood flow reserve. (A–C) End-systolic perfusion by myocardial contrast echocardiography in apical four- chamber view. (A) Mild perfusion defect of LV part segments (arrows) before high-MI flash impulse. (B) Significant reduction in perfusion in the sixth sec post flash (arrows) before adenosine infusion. (C) Three minutes after adenosine infusion showing significant reduction in perfusion in the third sec post flash (arrows). (D,E) Coronary blood flow velocity spectrum of the mid left anterior descending artery (LAD). (D) Before adenosine infusion. (E) At the third minute after adenosine infusion. Reduced blood flow is seen with minimal systolic blood flow (white arrow), and increased diastolic blood flow resistance (yellow arrow).”

The original version of this article has been updated.

